# Relationship between Dietary Macronutrients Intake and the ATHLOS Healthy Ageing Scale: Results from the Polish Arm of the HAPIEE Study

**DOI:** 10.3390/nu14122454

**Published:** 2022-06-14

**Authors:** Urszula Stepaniak, Maciej Polak, Denes Stefler, Magdalena Kozela, Martin Bobak, Albert Sanchez-Niubo, José Luis Ayuso-Mateos, Josep Maria Haro, Andrzej Pająk

**Affiliations:** 1 Department of Epidemiology and Population Studies, Jagiellonian University Medical College, 31-066 Krakow, Poland; maciej.1.polak@uj.edu.pl (M.P.); m.kozela@uj.edu.pl (M.K.); andrzej.pajak@uj.edu.pl (A.P.); 2 Department of Epidemiology and Public Health, University College London, London WC1E 6BT, UK; denes.stefler@ucl.ac.uk (D.S.); m.bobak@ucl.ac.uk (M.B.); 3 Centro de Investigación Biomédica en Red de Salud Mental, CIBERSAM, 28029 Madrid, Spain; asanchezniubo@ub.edu (A.S.-N.); joseluis.ayuso@uam.es (J.L.A.-M.); josepmaria.haro@sjd.es (J.M.H.); 4 Department of Social Psychology and Quantitative Psychology, University of Barcelona, 08035 Barcelona, Spain; 5 Department of Psychiatry, Universidad Autónoma de Madrid, 28029 Madrid, Spain; 6 Research, Innovation and Teaching Unit, Parc Sanitari Sant Joan de Déu, 08830 Sant Boi de Llobregat, Spain

**Keywords:** macronutrients, healthy ageing, scale, Central and Eastern Europe

## Abstract

Background: Despite extensive research, our knowledge on the relationship between nutrition and healthy ageing is limited. The aim of this study was to evaluate the associations between the intake of macronutrients and a single measure of healthy ageing (ATHLOS Healthy Ageing Scale). Methods: A cross-sectional analysis was performed using data from 9906 randomly selected citizens of Krakow (Poland) who were 45–69 years of age and participants of the Health, Alcohol and Psychosocial factors in Eastern Europe (HAPIEE) study. Macronutrient intake was evaluated using a food frequency questionnaire. ATHLOS Health Ageing Scale was estimated from 41 variables in pooled data from 16 cohorts. Standardized beta coefficients were estimated using multivariable linear regression models. Results: In multivariable adjusted models, there were significant positive associations between the ATHLOS Healthy Ageing Scale score and intake of protein (b = 0.030, 95% CI 0.001; 0.059 in men; b = 0.056, 95% CI 0.027; 0.085 in women), monounsaturated fatty acids (MUFA) (b = 0.042, 95% CI 0.013; 0.071 in men; b = 0.035, 95% CI 0.006; 0.064 in women), polyunsaturated fatty acids (PUFA) (b = 0.053, 95% CI 0.024; 0.082 in men; b = 0.063, 95% CI 0.034; 0.092 in women), and omega-3 PUFA (b = 0.031, 95% CI 0.002;0.060 in men; b = 0.054, 95% CI 0.026; 0.082 in women). Carbohydrate intake was inversely associated with the ATHLOS Healthy Ageing Scale in women. Total fat intake was positively associated with the ATHLOS Healthy Ageing Scale in men. Conclusions: A number of nutrients were associated with the healthy ageing score, suggesting that dietary habits may play an important role in healthy ageing. Further research in other settings and with a prospective design is strongly warranted.

## 1. Introduction

The population in the European Region of the WHO (World Health Organization) is ageing rapidly [1]. In 2020, 20.6% of the EU’s population was aged 65 and over, and this proportion is expected to increase substantially over the coming decades [2]. An ageing population imposes a significant burden on health and social care systems; therefore, initiatives that help people remain healthy in old age are important for public health [3].

Healthy ageing is defined as an “ongoing process of developing and maintaining the functional ability that enables well-being in older age” [4]. It is a process that everyone experiences differently, since it involves interactions between biological and molecular mechanisms, and with the environment [5,6]. The ageing phenotype appears to be the result of the gradual accumulation of damage to macromolecules, such as DNA, proteins and lipids, which may occur due to inflammation, or metabolic and oxidative stress [7].

Previous evidence also suggests that nutrition may modulate the ageing process [7,8]. For example, obesity is related to shorter lifespan, while adherence to healthy eating patterns, such as the Mediterranean dietary pattern, is associated with longevity and reduced risk of age-related diseases [7]. A diet with adequate nutrient content is essential for physical and mental wellbeing throughout the entire lifespan, until the later stages of life [9]. A poor-quality diet may contribute to malnutrition, which was found to be highly prevalent in Poland’s elderly population, and its consequences such as nutrient deficiencies, wasting, overweight and obesity [10,11]. Nutrition could potentially modify the incidence of many non-communicable chronic diseases through changes in biochemical and epigenetic factors [12].

Protein, carbohydrates and fats are primary energy sources. Protein is a major regulator of muscle protein metabolism [13]. Adequate protein supply may slow age-related muscle loss, which is related to declining physical function [14,15]. Omega-3 PUFA, with their anti-inflammatory properties, may have a beneficial effect on chronic low-grade inflammation associated with ageing [16]. It is suggested that omega-3 PUFA have a protective effect on skeletal muscle growth and regeneration, and on brain ageing and cognitive functions [16,17].

In previous studies concerning the relationship between dietary macronutrients and ageing, various ageing-related concepts and indices were used (e.g., frailty, sarcopenia, successful ageing as lack of diseases, physical or cognitive functioning) but the results seemed to be inconclusive. Low protein and low fat intake were related with prevalence of frailty and sarcopenia [18,19,20]. In some prospective studies, dietary protein intake was related to better ageing-related indices [15,21,22,23], but not in all [24,25]. The impact of total intake of fat or carbohydrates in prospective observations was not significant [24,25,26]. One of the reasons for the ambiguity of the results obtained may be the lack of a tool that adequately tackled the complex concept of healthy ageing. Most of the research to date has examined only some of the characteristics of healthy ageing. No studies assessing the association between consumption of macronutrients and the universal indicator which broadly captures a person’s healthy ageing were found. In our analysis, we used the ATHLOS Healthy Ageing Scale, developed in response to WHO’s emphasis on the development of complex measures of healthy ageing. This is a novel tool which, due to its high reliability, global representativeness and inverse association with mortality, has the potential to contribute to worldwide research on healthy ageing [4,27].

The eating habits, lifestyle and socio-economic circumstances of Eastern Europe differ from those in Western countries, where research on the associations of nutrients with age-related health problems was mainly conducted. Economic transformation in CEE countries in the 1990s was associated with changes in terms of accessibility to foods, and over the next couple of decades, changes in food consumption were mostly beneficial; i.e., consumption of fruit and polyunsaturated fat had risen, whereas consumption of saturated fat had fallen [28]. Despite this improvement, the diet quality of adults in Poland is considered low [29,30]. In a 14-year prospective observation, it was shown that high adherence to a traditional Eastern European diet was associated with a higher risk of all-cause mortality (HR 1.31; 95% CI 1.04–1.65) [31]. In the Polish population at ages of 65 years or above (POLSENIOR study), the frequency of malnutrition was 7.5% and the risk of development of malnutrition was 38.9% [12]. On the other hand, the prevalence of overweight or obesity (body mass index (BMI) >25 kg/m^2^) in men and women at age 65–75 years is very common (about 80%) [32].

Quantitative assessment of the relationship between basic dietary components and comprehensive healthy ageing score would broaden the perspective of public health challenges in ageing populations of CEE. Therefore, the aim of the study was to evaluate the association between macronutrient intakes (i.e., protein, carbohydrates, fiber, total fat and types of fatty acids: saturated fatty acids (SFA), MUFA, PUFA, omega-6 PUFA, omega-3 PUFA, trans fats) and healthy ageing, assessed by the ATHLOS Healthy Ageing Scale, in an urban population of Poland.

## 2. Materials and Methods

This cross-sectional study was carried out in the framework of the HAPIEE study. The HAPIEE study is a multicenter prospective cohort study that aims to investigate psychosocial and dietary determinants of cardiovascular diseases (CVD) and other chronic conditions in Central and Eastern Europe. Details of the study design and methods have been published elsewhere [33]. The present analysis was conducted within the Polish arm of the HAPIEE study and included a random sample of Krakow residents. At baseline, in 2002–2005, a sample of 10,728 men and women aged 45–69 years, stratified by gender and 5-year age groups, was selected from the Krakow population register. The response rate was 61% [33]. Participants were first visited at home to complete a standard questionnaire and then invited to a clinic for examination.

A total of 10,002 participants had complete data on macronutrient intake and had valid data on the ATHLOS Healthy Ageing Scale. Further, for the current analysis, we excluded 96 individuals with extreme values of energy intake: <500 kcal/day/>4500 kcal/day in women; <800 kcal/day/>5000 kcal/day in men, resulting in a final sample size of 9906 participants (4820 men and 5086 women) included into the analysis.

The HAPIEE study was approved by the ethics committees at University College London and at the Jagiellonian University Medical College. All participants gave their written informed consent.

## 3. Measurements of Healthy Ageing

Individual healthy ageing was assessed using the ATHLOS Healthy Ageing Scale. This is a novel tool developed by the ATHLOS (Ageing Trajectories of Health-Longitudinal Opportunities and Synergies) consortium [4]. It was constructed using harmonized data from 16 cohorts from 38 different countries on six continents. The scale was based on 41 biopsychosocial aspects of health and functioning since it covers domains of vitality (pain, energy, etc.), sensory (vision and hearing), locomotion (stooping, kneeling, lifting, climbing stairs, getting up from sitting down, walking, etc.), cognition (memory, immediate and delayed recall, orientation in time, etc.), and activities of daily living (getting in or out of bed, bathing, getting dressed, using the toilet, eating, shopping, preparing meals, housework, etc.) that imply interaction with the individual’s environment [5].

An item response theory (IRT) model was used to develop the scale, which indicated adequate goodness of fit as a unidimensional measure and high reliability. The validity of the scale against sociodemographic, health factors and mortality has shown that this score corresponds well with health status and could be useful in international ageing studies [4]. Details on the construction of the scale and its performance have been widely presented and discussed in earlier publications [4,5]. Scale values follow a normal distribution of a mean of 50 and a standard deviation of 10 where higher values mean better healthy ageing. The HAPIEE study was one of the studies included in the harmonized ATHLOS database.

## 4. Measurements of Macronutrient Intake

Individual dietary intake of macronutrients and energy was calculated based on a standard food frequency questionnaire (FFQ) [29]. FFQ consisted of 148 food and drink items. An instruction manual that included photographs to facilitate the estimation of portion sizes was used. Participants were asked how often, on average, they had consumed that amount of a particular food during the last 3 months, with nine responses ranging from “six or more times per day” to “never or less than once per month”. Moreover, participants were asked to include additional foods and frequency of consumption by manual entry. Then, frequency of food consumption was converted into daily food consumption, and nutrient and energy intakes were calculated using the McCance and Widdowson food composition tables [29]. In this analysis, intake data regarding total energy (kcal/day), macronutrients (protein, carbohydrates, fiber, total fat in g/day), types of fatty acids (saturated fatty acids, SFA; monounsaturated fatty acids, MUFA; polyunsaturated fatty acids, PUFA; trans fatty acids) in g/day, and types of PUFA (omega-3 and omega-6) were calculated.

## 5. Potential Confounders

At baseline examination, a standard questionnaire was used to collect data on age, gender, education (categorized into two groups: university, secondary or lower), marital status (married/cohabited or single/widowed/divorced), smoking (three groups: current smoker, ex-smoker, non-smoker), leisure time physical activity (three groups: 0 min./week; 1–149 min./week; ≥150 min./week), and history of CVD (self-reported history of myocardial infarction or stroke yes/no). Based on standard measurements of body weight and height, body mass index (BMI, kg/m^2^) was calculated. Data on BMI was available for 8724 respondents who took part in a clinic examination. There were some missing data in confounder variables, i.e., education (0.1%), marital status (0.2%), smoking (0.3%), history of CVD (0.8%), physical activity (5.1%), and BMI (11.9%).

## 6. Statistical Analysis

The quantitative variables were presented as mean and standard deviation (SD). The Shapiro–Wilk test was used to test the assumption of normal distribution. The continuous variables were compared between groups using the t-test for independent samples. Categorical variables were described using percentages and compared using the χ^2^ test. Dietary intakes of macronutrients were adjusted for total energy intake using the residual method [34]. Pearson’s correlation coefficient was used to examine the associations between macronutrient intake and the ATHLOS Healthy Ageing Scale. Multivariable linear regression models were conducted to determine which of the investigated macronutrients were associated with the ATHLOS Healthy Ageing Scale, after adjustment for potential confounders. Results were presented as beta standardized coefficient (per increase of 1 SD) with a 95% CI. In the fully adjusted model, we used the following potential confounders: age, education, marital status, smoking, BMI, physical activity, history of CVD, and total energy intake. Because of the two-stage nature of the baseline examination, the participation rate for the clinical examination was lower than for the interview. Thus, the number of persons included in the final multivariate model was lower (by approximately 12%), as the sample was restricted to participants without missing data on any of the covariates. All results are presented by sex because exposure and outcome were significantly different in men and women. Statistical analyses were performed using IBM SPSS Statistics for Windows, Version 27.0. Armonk, NY, USA: IBM Corp. *p*-values < 0.05 were accepted as statistically significant.

## 7. Results

Baseline characteristics for 9906 subjects are presented in Table 1 by gender. The mean age of participants was 57.9 (SD = 6.95) years in men and 57.4 (SD = 6.98) in women. The mean of the ATHLOS Healthy Ageing Scale score was higher in men (51.3 points SD = 8.90) than in women (47.5 points SD = 8.98). The proportions of participants with university education, who were married, who were currently smoking or who had smoked in the past were higher in men than in women. Physical activity was lower in women. Frequency of history of CVD was higher in men. Mean BMI was slightly higher in women. Intake of total energy, protein, and fat was higher in men, while consumption of carbohydrates was higher in women.

Pearson’s correlation coefficients showing associations between macronutrient intake and the ATHLOS Healthy Ageing Scale scores are presented in Table 2. A positive correlation between the ATHLOS Healthy Ageing Scale scores and total fat, MUFA, PUFA was observed in both genders (r = 0.046; r = 0.039; r = 0.048 and r = 0.042; r = 0.038; r = 0.072, respectively, in men and women). In women, there was a positive correlation between the ATHLOS Healthy Ageing Scale scores and intake of protein (r = 0.044) and omega-3 PUFA (0.048). In men, but not in women, negative correlations between the ATHLOS Healthy Ageing Scale scores and carbohydrates (r= −0.042) and trans fatty acids (−0.029) were shown.

Results of the multivariable adjusted linear regression model are presented in Table 3. After adjustment for age, education, marital status, smoking, BMI, physical activity, history of CVD, and total energy intake, in both men and women, there were significant positive associations between intake of protein (b = 0.030, 95% CI 0.001; 0.059 in men; b = 0.056, 95% CI 0.027; 0.085 in women), MUFA (b = 0.042, 95% CI 0.013; 0.071 in men; b = 0.035, 95% CI 0.006; 0.064 in women), PUFA (b = 0.053, 95% CI 0.024; 0.082 in men; b = 0.063, 95% CI 0.034; 0.092 in women), omega-3 PUFA (b = 0.031, 95% CI 0.002; 0.060 in men; b = 0.054, 95% CI 0.026; 0.082 in women) and the ATHLOS Healthy Ageing Scale scores. The strongest relationship was observed between PUFA and ATHLOS Healthy Ageing Scale in men and women.

Total fat intake was positively associated with the ATHLOS Healthy Ageing Scale score in men only (b = 0.042, 95% CI 0.013; 0.071). Carbohydrate intake was negatively associated with the ATHLOS Healthy Ageing Scale score in women only (b= −0.030, 95% CI −0.059; −0.001). There was no significant association between intake of: SFA, omega-6 PUFA, trans fatty acids, fiber and the ATHLOS Healthy Ageing Scale.

## 8. Discussion

In this urban Polish population-based cohort, higher consumption of protein, MUFA, omega-3 PUFA and PUFA was associated with healthier ageing, as measured by the ATHLOS Healthy Ageing Scale, in both men and women. Additionally, in women only, higher consumption of carbohydrates was associated with lower healthy ageing score, indicating worse ageing. In men, higher consumption of total fat was related to better healthy ageing score.

Our results indicate the beneficial effect of protein intake in terms of ageing. It is known that protein requirements increase with age. Dietary protein intake stimulates muscle protein synthesis, which leads to improved muscle mass, strength and function over time. Increased muscle mass, strength and function are related to improved health outcomes in older individuals [35]. The positive association between protein intake and the ATHLOS Healthy Ageing Scale in our cohort is in line with studies which investigated the relationship of diet with other measures of ageing [15,18,19,20,21,22,36,37]. In cross-sectional studies, lower dietary protein intake was associated with frailty in elderly Japanese women [36], in adults in Spain [19] and with sarcopenia in elderly Belgians [18], or in Australian men [20]. In the NHANES study of 11,680 adults over 51 years old, lower protein intake was associated with more functional limitations [37]. In a prospective observation of 24,417 women aged 65–79 years within the Women’s Health Initiative study, protein intake was inversely associated with risk of frailty [21].

In a 6-year prospective study of initially well-functioning adults aged 70–79 years in the US population, lower protein intake was associated with increased risk of mobility limitation [22]. Results of a 5-year prospective cohort study of 722 adults aged 85+ in the United Kingdom showed that protein intake was associated with better disability trajectories [15]. However, in some prospective studies, protein intake was not related to frailty [24,25].

In our study, we found an inverse association between carbohydrate intake and healthy ageing in women, but not in men. In other studies, carbohydrate intake was not significantly associated with successful ageing (defined as the absence of disability, depressive symptoms, cognitive impairment, respiratory symptoms, and chronic diseases (e.g., cancer and coronary artery disease)) in a 10-year prospective study in Australia [26]. Similarly, there was no association between intake of carbohydrates and frailty in a 11-year observation of participants of the Rotterdam Study [25], or in men in the US population [24].

The significant unfavorable effect of carbohydrates in women in our study may be related to higher intake of carbohydrates in women than in men. Carbohydrates are a heterogeneous group that includes, among others, sugar and complex carbohydrates. In the population of Poland, it was shown that, in most of the adult population, consumption of sugar is considerably above the recommendation, whereas consumption of complex carbohydrates is low and most of the population do not meet the recommendations [29,30,38]. Refined grains and sweets were found to be the main sources of carbohydrates in Polish women [39]. High intake of carbohydrates may promote obesity and its consequences. Additionally, high intake of sugar is related to cognitive impairment [40]. Moreover, results of the meta-analysis of cohort studies on carbohydrate intake and mortality showed that high consumption, compared to moderate, was related with increased total mortality (RR = 1.23; 95% CI: 1.11–1.36) [41].

In our study, higher intake of total fat was associated with better ageing in men but not in women. Consumption of fat was higher in men than in women. Little is known on total fat and ageing indices. For example, total fat intake was not significantly associated with frailty in an 11-year observation of middle-aged and older adults in the Rotterdam Study [25], as well as in the cohort study of the US men aged 65+ [24].

Total fat is a heterogeneous group of components with different health effects. Among categories of fatty acids, we found a significant association between MUFA, omega-3 PUFA, PUFA and ATHLOS Healthy Ageing Scale in men and women.

MUFA are known to be beneficial in the prevention of inflammation, free radical production, or even neurodegeneration [42]. Our result is in line with other studies which indicated that MUFA intake is associated with a lower risk of frailty [43]. MUFA are a central part of the Mediterranean diet, which is well known as a healthy diet, resulting in lower mortality [44] and lower CVD incidence and CVD mortality [45] as well as lower risk of dementia [46]. It may be expected that effects on mortality will be reflected by increased longevity.

The relationship between PUFA and the ATHLOS Healthy Ageing Scale in our cohort resulted from a significant relationship with omega-3 PUFA but not omega-6 PUFA consumption. In the population of Poland, it was shown that intake of PUFA is low and only 16% of adults meet the recommendations [29].

The beneficial effect of omega-3 PUFA shown in our study is in line with other studies which showed their beneficial effects on health status. Omega-3 PUFA are known to reduce inflammation, which is involved in the development of ageing and age-related disease such as CVD. Results of the recent meta-analysis of 17 prospective cohort studies examining the associations between blood omega-3 fatty acid levels and risk for mortality showed that in the highest quintile, the risk of all-cause, CVD and cancer mortality was significantly lower by about 15–20% compared to the lowest quintile [47]. Additionally, due to effects on endothelial function, impacts on cerebral blood flow, and slowing degradation of neural tissue [48], omega-3 PUFA might have a positive effect on cognitive function [49]. Omega-3 PUFA have been suggested to have positive age-related effects on anabolic resistance and, therefore, enhance gains in muscle mass in older adults [18,50,51]. Intake of fish rich in omega-3 PUFA was associated with reduced incidence of frailty during 3.5 years of follow-up in the Seniors-ENRICA study [52]. However, in some studies, omega-3 PUFA were not significantly related to frailty [51,53]. Results of the systematic review on RCT studies on dietary interventions to increase intake of omega-3 PUFA suggest that increasing omega-3 has little or no effect on functional status [54].

Between-sex differences in the relation between nutrition and ageing were not found in the study in which the relations between nutrition and frailty were similar after stratification by sex [25]. However, in that study, intake of carbohydrates or fat was not associated with frailty. Other studies did not focus on between-sex differences [15,22,23,26] or were conducted only in women [21] or only in men [24]. The possible explanation of the between-sex differences in our study could partially be explained by the differences in the consumption of fat and carbohydrates between men and women. In an earlier study on animals, a low-protein diet was suggested to be beneficial for people under the age of 65 years, while a high-protein diet was beneficial for those over the age of 65 years [55]. This was not confirmed in our study in the analysis after stratification by age group.

Our study has several strengths. Using the ATHLOS Healthy Ageing Scale allowed us to assess participants’ ageing in a comprehensive manner, rather than through fragmented, ageing-related health outcomes. The fact that we used data from a large Polish cohort is another strength, since any evidence on the examined association in the Central and Eastern European region is particularly rare.

This study also has some important limitations. Firstly, due to the cross-sectional study design, we cannot exclude the possibility of reverse causation and that certain ageing-related conditions may influence participants’ dietary habits. In order to clarify this question, further research with a prospective study design is recommended. Next, the study consisted of only an urban population; hence, the results may not be generalizable to other, rural populations, or the Polish population as a whole. Additionally, despite the comprehensive information on lifestyle factors and careful adjustment for covariates, there is still the possibility of residual confounding by unmeasured or incompletely controlled variables that might be related to both diet and health in ageing. Further, we could not include in the analysis the use of omega-3 PUFA supplements as these data were not available. Due to the lack of data on the consumption of the source of the protein (plant vs. animal), it was also not possible to assess the relationship between the source of the protein and health in ageing.

## 9. Conclusions

The results of this cross-sectional study indicate that dietary habits may play an important role in healthy ageing. Public health nutritional interventions in Poland are clearly needed in order to improve the quality of the diet, in this way contributing to healthier ageing of the population.

## Figures and Tables

**Table 1:** Baseline characteristics of participants of the HAPIEE Krakow study by sex.

Characteristics	Men *n* = 4820	Women *n* = 5086	*p* *
Age [years], mean (SD)	57.9 (6.95)	57.4 (6.98)	<0.001
the ATHLOS Healthy Ageing Scale, mean (SD)	51.3 (8.90)	47.5 (8.98)	<0.001
Education (%)			<0.001
middle or lower	69.8	72.9	
university	30.2	27.1	
Marital status (%)			<0.001
single, widowed, divorced	13.3	33.1	
married, cohabited	86.7	66.9	
Smoking status (%)			<0.001
current smoker	36.0	28.7	
ex-smoker	36.3	20.9	
Non-smoker	27.7	50.4	
Physical activity groups (%)			0.03
0 (min/week)	28.9	30.4	
1–149 (min/week)	13.7	12.0	
>150 (min/week)	57.4	57.5	
History of CVD (%)			<0.001
no	89.7	96.1	
yes	10.3	3.9	
BMI [kg/m^2^], mean (SD)	28.0 (4.04)	28.3 (5.07)	<0.001
Energy [kcal], mean (SD)	2255.5 (680.69)	2043.2 (612.76)	<0.001
Protein [g], mean (SD)	94.6 (13.39)	92.6 (13.81)	<0.001
Carbohydrates [g], mean (SD)	239.4 (38.90)	255.2 (38.61)	<0.001
Fiber [g], mean (SD)	17.6 (4.84)	19.3 (5.55)	<0.001
Total fat [g], mean (SD)	76.5 (14.26)	72.7 (14.01)	<0.001
SFA [g], mean (SD)	33.8 (6.96)	32.8 (6.92)	<0.001
MUFA [g], mean (SD)	28.6 (6.46)	26.2 (6.14)	<0.001
PUFA [g], mean (SD)	11.6 (2.78)	11.3 (2.88)	<0.001
Omega-6 PUFA [g], mean (SD)	4.3 (2.01)	4.0 (1.95)	<0.001
Omega-3 PUFA [g], mean (SD)	0.8 (0.42)	0.8 (0.40)	<0.001
Trans fatty acids [g], mean (SD)	2.5 (0.86)	2.4 (0.78)	<0.001

* χ^2^ test for categorical variables and t-test for continuous variables. SFA—saturated fatty acids; MUFA—monounsaturated fatty acids; PUFA—polyunsaturated fatty acids; CVD—cardiovascular diseases; BMI—body mass index.

**Table 2:** Correlation between the ATHLOS Healthy Ageing Scale and macronutrient intake.

Macronutrients	Men		Women	
	r	*p*	r	*p*
Protein	−0.007	0.643	0.044	0.002
Carbohydrates	−0.042	0.003	−0.012	0.409
Fiber	−0.024	0.092	0.009	0.511
Total fat	0.046	0.001	0.042	0.003
SFA	0.042	0.004	0.022	0.117
MUFA	0.039	0.007	0.038	0.007
PUFA	0.048	0.001	0.072	<0.001
Omega-6 PUFA	−0.008	0.601	0.024	0.091
Omega-3 PUFA	0.012	0.385	0.048	0.001
Trans fatty acids	−0.029	0.041	−0.008	0.577

**Table 3:** Association between macronutrient intake and the ATHLOS Healthy Ageing Scale—results of the multivariable linear regressions.

Macronutrients	Men			Women		
	B *	95% CI *	*p*	B *	95% CI *	*p*
Protein						
Model 1	0.002	−0.025; 0.029	0.886	0.045	0.019; 0.072	<0.001
Model 2	0.010	−0.017; 0.037	0.494	0.038	0.011; 0.065	0.005
Model 3	0.030	0.001; 0.059	0.040	0.056	0.027; 0.085	<0.001
Carbohydrates						
Model 1	−0.005	−0.033; 0.023	0.735	0.012	−0.014; 0.039	0.360
Model 2	−0.014	−0.041; 0.013	0.333	−0.017	−0.044; 0.010	0.212
Model 3	−0.014	−0.043; 0.015	0.338	−0.030	−0.059; −0.001	0.046
Fiber						
Model 1	0.015	−0.012; 0.043	0.278	0.024	−0.003; 0.051	0.076
Model 2	0.005	−0.023; 0.033	0.731	0.004	−0.024; 0.031	0.799
Model 3	0.024	−0.006; 0.053	0.115	0.009	−0.020; 0.037	0.556
Total fat						
Model 1	0.015	−0.012; 0.043	0.280	0.011	−0.016; 0.038	0.430
Model 2	0.051	0.024; 0.078	<0.001	0.021	−0.006; 0.048	0.135
Model 3	0.042	0.013; 0.071	0.005	0.027	−0.002; 0.056	0.065
SFA						
Model 1	0.016	−0.012; 0.043	0.260	−0.001	−0.027; 0.026	0.960
Model 2	0.045	0.018; 0.072	0.001	0.003	−0.024; 0.030	0.823
Model 3	0.027	−0.002; 0.056	0.069	−0.002	−0.031; 0.027	0.896
MUFA						
Model 1	0.001	−0.022; 0.034	0.680	0.003	−0.024; 0.030	0.081
Model 2	0.047	0.020; 0.074	0.001	0.023	−0.004; 0.050	0.096
Model 3	0.042	0.013; 0.071	0.005	0.035	0.006; 0.064	0.018
PUFA						
Model 1	0.034	0.007; 0.062	0.020	0.051	0.024; 0.077	<0.001
Model 2	0.042	0.015; 0.069	0.002	0.047	0.020; 0.074	0.001
Model 3	0.053	0.024; 0.082	<0.001	0.063	0.034; 0.092	<0.001
Omega-6 PUFA						
Model 1	−0.024	−0.052; 0.003	0.080	0.013	−0.013; 0.040	0.330
Model 2	−0.008	−0.035; 0.019	0.555	0.021	−0.006; 0.048	0.127
Model 3	−0.005	−0.034; 0.024	0.718	0.023	−0.005; 0.051	0.119
Omega-3 PUFA						
Model 1	0.006	−0.022; 0.033	0.680	0.044	0.017; 0.071	0.001
Model 2	0.015	−0.012; 0.042	0.274	0.041	0.014; 0.068	0.002
Model 3	0.031	0.002; 0.060	0.035	0.054	0.026; 0.082	<0.001
Trans fatty acids						
Model 1	−0.035	−0.063; −0.008	0.010	−0.017	−0.044; 0.009	0.210
Model 2	−0.015	−0.042; 0.012	0.282	−0.014	−0.041; 0.013	0.301
Model 3	−0.012	−0.041; 0.017	0.393	−0.005	−0.033; 0.023	0.736

* beta standardized coefficients reflect the difference in outcome per 1 SD increase in the macronutrient intakes. Model 1—adjusted to: age. Model 2—adjusted to: age, education, marital status, smoking, physical activity, energy intake. Model 3—adjusted to: age, education, marital status, smoking, physical activity, energy intake, BMI, history of CVD.
